# Changing the System - Major Trauma Patients and Their Outcomes in the NHS (England) 2008–17

**DOI:** 10.1016/j.eclinm.2018.07.001

**Published:** 2018-08-05

**Authors:** Christopher G. Moran, Fiona Lecky, Omar Bouamra, Tom Lawrence, Antoinette Edwards, Maralyn Woodford, Keith Willett, Timothy J. Coats

**Affiliations:** a University of Nottingham, Derby Rd, Nottingham NG7 2UH, UK; b Centre for Urgent and Emergency Care REsearch (CURE), Health Services Research Section, School of Health and Related Research, University of Sheffield, S1 4DA, UK; c Trauma Research and Audit Network, University of Manchester, 3rd Floor Mayo Building, Salford Royal NHS Foundation Trust, Salford M6 8HD, UK; d Kadoorie Research Centre, Nuffield Department of Orthopaedics, Rheumatology and Musculoskeletal Sciences, University of Oxford, Headley Way, Headington, Oxford OX3 9DU, UK; e University of Leicester, University Road, Leicester LE1 7RH, UK

**Keywords:** Major trauma, Wounds and injuries, Trauma systems

## Abstract

**Background:**

Trauma care in England was re-organised in 2012 with ambulance bypass of local hospitals to newly designated Major Trauma Centres (MTCs). There is still controversy about the optimal way to organise health series for patients suffering severe injury.

**Methods:**

A longitudinal series of annual cross-sectional studies of care process and outcomes from April 2008 to March 2017. Data was collected through the national clinical audit of major trauma care. The primary analysis was carried out on the 110,863 patients admitted to 35 hospitals that were ‘consistent submitters’ throughout the study period. The main outcome was longitudinal analysis of risk adjusted survival.

**Findings:**

Major Trauma networks were associated with significant changes in (1) patient flow (with increased numbers treated in Major Trauma Centres), (2) treatment systems (more consultant led care and more rapid imaging), (3) patient factors (an increase in older trauma), and (4) clinical care (new massive transfusion policies and use of tranexamic acid). There were 10,247 (9.2%) deaths in the 110,863 patients with an ISS of 9 or more. There were no changes in unadjusted mortality. The analysis of trends in risk adjusted survival for study hospitals shows a 19% (95% CI 3%–36%) increase in the case mix adjusted odds of survival from severe injury over the 9-year study period. Interrupted time series analysis showed a significant positive change in the slope after the intervention time point of April 2012 (+ 0.08% excess survivors per quarter, p = 0.023), in other words an increase of 0.08 more survivors per 100 patients every quarter.

**Interpretation:**

A whole system national change was associated with significant improvements in both the care process and outcomes of patients after severe injury.

**Funding:**

This analysis was carried out independently and did not receive funding. The data collection for the national clinical audit was funded by subscriptions from participating hospitals.

**Research in context**

**Evidence before this study**

There is a large but controversial literature on the effect of trauma systems, as controlled trials are not possible. Previous studies have been conducted on a local or regional level and most have focused on selected major trauma centre patients, with associated high risk of bias.

**Added value of this study**

This is the first analysis of the effects on survival following the introduction of systematic trauma care on a national basis in a health system that provides universal healthcare coverage. Trauma care reorganisation was associated with a significant, 19% increase in the odds of survival following major trauma in the first 5 years after inception. This change continued to be present in sensitivity analyses which explored potential bias from missing data and changing patterns of data submission to the registry.

**Implications of all the available evidence**

Observational studies are likely to be the highest form of evidence available for healthcare planners to use as a basis for future decisions about trauma care. The evidence suggests that the best approach is re-organisation to create inclusive trauma networks with tiered hospitals and ambulance service bypass to the most appropriate centre.

Alt-text: Unlabelled Box

## 1 Introduction

Trauma remains one of the commonest causes of death and disability worldwide [1]. Evidence from Germany [2], Australia [3], [4] and the USA [5], [6] shows that trauma care improves with rationalisation of trauma systems with best results produced when care within a region is provided by a network of hospitals with a Major Trauma Centre (MTC) at the hub. In America, states with trauma systems have a 9% lower crude mortality rate than states without organised systems of trauma care [7], although there are many potential confounders. Using risk adjustment for potential confounders, the introduction of a trauma system in the Australian state of Victoria was associated with a relative reduction in mortality of 38% over a five-year period (Adjusted Odds Ratio = 0.62; 95% CI 0.48 to 0.80) [8].

In England, trauma is the commonest cause of death in those under 40 years, with survivors often suffering long-term disability [9]. The National Audit Office estimates that there are 20,000 cases of major trauma each year in England with 5400 deaths [10]. The NCEPOD 2007 report “Trauma, who cares?” also identified serious failings in the organisation of trauma care in England [11]. Since the inception of the National Health Service (NHS) in 1948, the emergency care system had been based upon the ambulance service transporting the patient to the nearest Accident and Emergency Department, irrespective of the capability of the hospital to provide resuscitation and definitive care. The Trauma Audit and Research Network (TARN) had identified great variation in the standard of care in England and comparative studies showed that the outcome following trauma did not meet the standards of other countries [12]. The NCEPOD 2007 report suggested that almost 60% of major trauma patients received a standard of care that was less than good practice with avoidable deaths still occurring. This led the National Audit Office to recommend the development of regional trauma networks in England.

Following on from this recommendation, the NHS reorganisation created a series of Regional Networks. In England there are now 27 designated Major Trauma Centres: 11 for adults and children, 10 for adults only, 5 for children only and 1 collaborative centre (a group of geographically close hospitals who collaborate to provide full MTC services). The London network started in April 2010 and the Trauma Networks across England started operating in April 2012. The additional services provided by Trauma Networks are funded by a “Best Practice Tariff”, which is an additional payment per patient of £1406 ($1887) for patients with an ISS of 9 to 15, or £2819 ($3783) for patients with an ISS of 16 or more (2018 prices). This is in addition to the usual patient funding and is only payed to MTCs (there is no additional funding for TUs).

Previous studies of the effect of introduction of trauma systems have looked at local or regional initiatives, usually based on local clinical enthusiasm or based on centres of excellence. This gives a difficulty in understanding the generalizability of the results as it is well known that improved outcomes driven by enthusiasts may not be reproduced when applied more widely. Previous work has often excluded the patients who were not identified in the trauma care system and therefore did not go to a Major Trauma Centre (MTC) and who may have had worse outcomes with treatment in units that had been ‘deskilled’ [13], [14]. The universal healthcare coverage provided by the NHS allows study of comprehensive national trauma registry data to evaluate the effects of the implementation of national regionalisation of trauma care on both the process of care and patient mortality. The use of TARN data to assess the impact of changes to the care of the severely injured was recommended in 2010 by the NHS Clinical Advisory Groups Report “Regional Networks for Major Trauma” and was planned as part of the reorganisation of trauma care.

## 2 Methods

The study design and reporting followed the STROBE guidance for observational studies [15]. Longitudinal data was collected by the Trauma Audit and Research Network (the national clinical audit for major trauma care) using a consistent methodology before, during and after the reorganisation of trauma care in England and North Wales. For this study, anonymised data from patients presenting between 1st April 2008 and 31st March 2017 was collated for analysis. The UK Health Research Authority Patient Information Advisory Group (PIAG) has given approval (Section 20) for research using anonymised TARN data.

The TARN database includes patients of any age who sustain injury resulting in: hospital admission > 72 h, critical care admission, transfer to a tertiary/specialist centre or death within 30 days. Isolated femoral neck or single pubic ramus fracture in patients > 65 years and simple isolated injuries are excluded. After study inclusion, a dataset of prospectively recorded variables covering demographics plus injury-related physiological, investigation, treatment and outcome parameters are collated using a standard web-based case record form by TARN hospital audit co-ordinators. Injury descriptions from imaging, operative and necropsy reports are submitted by TARN co-ordinators - all injuries are coded centrally using the Abbreviated Injury Scale, this enables calculation of the Injury Severity Score (ISS) [16]. Some patients meet TARN inclusion criteria with injury combinations of low severity – for example a simple zygomatic and closed forearm fracture (ISS = 8). It is implausible that the re-organisation of trauma care would impact on care or outcomes for these patients. Hence for this study TARN patients with an ISS < 9 were excluded. Patients subsequently transferred to non-TARN participating hospitals were excluded from the analysis, as outcome was unknown. Outcome in terms of survival or death was based on the assessment at discharge or 30 days, whichever occurred first. Traumatic Brain Injury (TBI) was defined as patients with a head region Abbreviated Injury Score (AIS) > 2 (i.e. abnormal CT brain scan or a clinically open/base of skull fracture). All other patients were regarded as not having sustained TBI.

Over the study period 98 English trauma receiving hospitals joined TARN for the first time increasing membership from 91 hospitals in 2008/9 to 169 hospitals in 2016/17, including all designated MTCs and Trauma Units. A subgroup of 35 hospitals with continuous TARN membership and patient submissions throughout the study period was identified and was defined as “consistent submitters” and the primary analyses describing trends in case-mix, care processes and outcomes were undertaken on this group.

It may be that the ‘constant submitter’ hospitals are different from the rest of the hospitals in England and Wales as they are self-defined by long term membership of the national trauma audit system. This might introduce bias that would impair the generalisability of any conclusions (for example they may be more willing to engage in the Trauma Network and so show more improvement than others, or they may already have such good outcomes that Network formation did not lead to change). We attempted to look for bias by repeating the analysis using data from all hospitals in England and Wales rather than just the ‘constant submitters’.

As missing values for Glasgow Coma Scale (GCS) are known to bias trauma outcome models [18], multiple imputations were performed for missing GCS values (assuming that the mechanism of missingness is at random). As missing data could also have caused bias we performed a sensitivity analysis by repeating the analysis including and excluding cases with missing data.

## 3 Analysis

The study size was determined by the number of eligible patients included in the TARN database. Patient demographic features, injury characteristics, patterns of care and crude mortality were described by year of presentation. To define the changes in care that took place trends in the yearly proportion of location of care and other categorical variables were examined using Chi-squared test for trend. A two-sided p value of < 0.05 was considered to be statistically significant. As small changes within such a large dataset give rise to ‘statistical significance’ only changes that were considered by the authors to also have ‘clinical significance’ were highlighted in the text (all data were presented in the tables).

To examine temporal trends in trauma outcome (mortality), the standard logistic regression modelling approach used in trauma outcome analysis [17] was employed. Survival to acute care discharge or 30 days (whichever is first) was used as the dependent variable, with year of treatment as an independent variable, allowing calculation of odds of survival for each financial year (1st April to 31st March) from 2009/10 to 2016/17 compared to the 2008/9 baseline. The effect of differing case mix between each year of attendance was adjusted for by specifying potential confounders (those used in the established trauma prognostic model) as covariates. The explanatory variables considered were: admission GCS, injury severity score (ISS) [16], age and an age/gender interaction, and comorbidity.

The primary analysis was performed on data from the group of ‘consistently submitting’ hospitals. Results were reported as case-mix adjusted and unadjusted yearly odds of death, with 95% confidence intervals, and plotted graphically. Further sensitivity analysis was conducted on just the cases with complete data and cases from all hospitals. The discrimination of logistic regression models was assessed using the area under the receiving operator curve (AUROC).

To further characterise any changes in risk adjusted survival over the study time period the quarterly W statistics were calculated for each consecutive 3 month period from April of each year using the conventional TARN method [19]. The W can be interpreted as the number of excess survivors per 100 patients (observed – expected given case mix). An interrupted time series analysis (ITS) around the introduction of the trauma networks in April 2012/13 was performed using the quarterly W on aggregate data with autoregressive regression to allow for autocorrelation. This technique can assess step changes in magnitude as well as changes in secular trends of a specified variable with units around a given point in time [20].

Statistical analyses were carried out in Stata version 14 (StataCorp, College Station, USA). Missing GCS values were imputed by chained equations implemented using the ICE Stata (StataCorp, College Station, USA) procedure.

## 4 Results

**Fig. 1:**
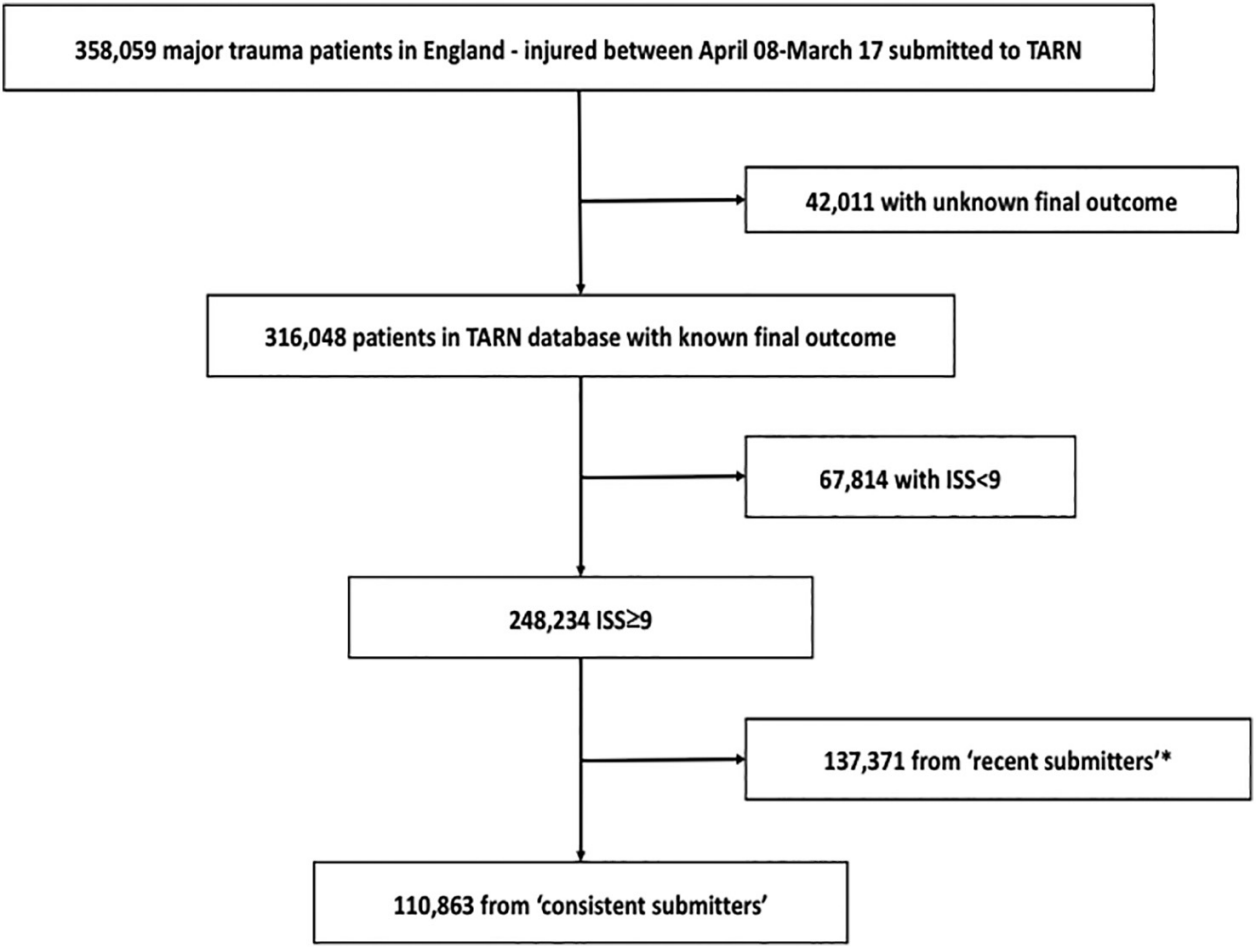
STROBE diagram identifying eligible study patients from TARN database in consistently submitting hospitals. *(Patients submitted from hospitals that began submitting after the study period commenced – these were combined with the study group in sensitivity analyses).

The process of identification of eligible patients is shown in Fig. 1. There were 20,605 (8.3%) deaths in the 248,234 patients with an ISS of 9 or more with known outcome, 10,247 (9.2%) in the 110,863 patients from hospitals who were ‘consistent submitters’.

There were changes in the patient characteristics (case mix) from 08/09 to 16/17 (Table 1). The median age of the patients increased significantly from 45 to 59 years (p = 0.0001), with the proportion of major trauma patients aged 65 years and over almost doubling from 22% to 42% (p < 0.0001). The yearly proportion of male patients fell significantly from 68% to 61% (p < 0.0001). With the increasing age over the study period, the proportion of patients with a co-morbid condition increased from 40% to 64% (p < 0.0001). The distribution of injuries (proportion with TBI/extracranial injuries – not mutually exclusive – at 35%/73%) and their median severity (median ISS = 16/median Emergency Department arrival GCS = 15) have not significantly changed over the study period. The annual proportion of patients injured by a fall from less than 2 m in height has significantly increased from 32% to 47% (p < 0.0001). The proportions injured by other mechanisms have all reduced slightly over time but the change is not significant within each sub-category. The annual number of major trauma patients with an ISS > 8 presenting to these consistently submitting hospitals increased 260% from 5338 to 19,197 over the study period, the increase being most marked in those injured by low falls. Similar findings are evident in the sensitivity analysis of patients from all hospitals (Table 3).

**Table 1:** Characteristics of the population from hospitals with consistent submissions by financial year 08/09–16/17 – patients with an ISS ≥ 9.

	2008/09	2009/10	2010/11	2011/12	2012/13	2013/14	2014/15	2015/16	2016/17	Total
Hospitals submitting to TARN	35	35	35	35	35	35	35	35	35	35
TARN cases	5338	6957	8626	9679	11,708	14,793	16,414	18,151	19,197	110,863
Age, median (IQR)	45 (25.8–63.2)	47.9 (27.2–66)	48.3 (28.1–66.3)	48.9 (28.2–67.7)	52.8 (31.2–72.7)	54.7 (33–75.1)	56.7 (34.6–76.7)	57.5 (35.2–77.9)	59 (36.5–78.5)	54.2 (32–74.5)
Age > 64, n (%)	1160 (21.7%)	1787 (25.7%)	2253 (26.1%)	2710 (28%)	3835 (32.8%)	5248 (35.5%)	6330 (38.6%)	7261 (40%)	8056 (42%)	38,640 (34.9%)
Male, n (%)	3621 (67.8%)	4613 (66.3%)	5633 (65.3%)	6377 (65.9%)	7340 (62.7%)	9276 (62.7%)	10,152 (61.8%)	11,271 (62.1%)	11,715 (61%)	69,998 (63.1%)
ISS, median (IQR)	14 (9–24)	16 (9–25)	16 (9–25)	16 (9–25)	14 (9–24)	14 (9–25)	14 (9–25)	16 (9–25)	16 (9–25)	16 (9–25)
First hospital GCS, median (IQR)	15 (14–15)	15 (14–15)	15 (14–15)	15 (14–15)	15 (14–15)	15 (14–15)	15 (14–15)	15 (14–15)	15 (14–15)	15 (14–15)
RTC, n (%)	1833 (34.3%)	2223 (32%)	2622 (30.4%)	3041 (31.4%)	3343 (28.6%)	4204 (28.4%)	4581 (27.9%)	4887 (26.9%)	5044 (26.3%)	31,778 (28.7%)
High fall, n (%)	874 (16.4%)	1099 (15.8%)	1405 (16.3%)	1489 (15.4%)	1737 (14.8%)	2256 (15.3%)	2521 (15.4%)	2616 (14.4%)	2730 (14.2%)	16,727 (15.1%)
Low fall, n (%)	1706 (32%)	2492 (35.8%)	3108 (36%)	3624 (37.4%)	5022 (42.9%)	6413 (43.4%)	7354 (44.8%)	8441 (46.5%)	9064 (47.2%)	47,224 (42.6%)
Other, n (%)	393 (7.4%)	463 (6.7%)	591 (6.9%)	635 (6.6%)	720 (6.1%)	882 (6%)	862 (5.3%)	956 (5.3%)	938 (4.9%)	6440 (5.8%)
Assault, n (%)	532 (10%)	680 (9.8%)	900 (10.4%)	890 (9.2%)	886 (7.6%)	1038 (7%)	1096 (6.7%)	1251 (6.9%)	1421 (7.4%)	8694 (7.8%)
Penetrating, n (%)	367 (6.9%)	419 (6%)	508 (5.9%)	563 (5.8%)	567 (4.8%)	605 (4.1%)	603 (3.7%)	811 (4.5%)	900 (4.7%)	5343 (4.8%)
TBI, n (%)	1887 (36.6%)	2472 (36.7%)	2915 (36%)	3166 (35.1%)	3515 (33%)	4483 (33.4%)	5012 (33.8%)	5655 (34.7%)	6073 (35%)	35,178 (34.6%)
Extracranial, n (%)	3650 (70.7%)	4804 (71.3%)	5869 (72.4%)	6584 (73%)	7830 (73.4%)	9827 (73.2%)	10,863 (73.2%)	11,752 (72.2%)	12,462 (71.8%)	73,641 (72.5%)
Comorbidity present, n (%)	2119 (39.7%)	3051 (43.9%)	4083 (47.3%)	4936 (51%)	6192 (52.9%)	8072 (54.6%)	9497 (57.9%)	11,417 (62.9%)	12,257 (63.8%)	61,624 (55.6%)

There were changes in the process of care over time (Table 2). Within the study sample of 35 consistently submitting hospitals, 15 (43%) were designated as Major Trauma Centres, which is higher than the national proportion of 16% (27 out of 169). In these ‘consistently submitting’ hospitals there was an increase in the proportion of patients having a Major Trauma Centre as an initial (53% to 72% p < 0.0001), or final (73% to 82% p < 0.0001) care destination. There has been an increase in time from injury to arrival at the first hospital (+ 30 min, p < 0.0001). The proportion of patients having CT imaging has increased from 50% to 72% (p < 0.0001).

**Table 2:** Care process in hospitals with consistent submissions by financial year 08/09–16/17 – patients with ISS ≥ 9.

	2008/09	2009/10	2010/11	2011/12	2012/13	2013/14	2014/15	2015/16	2016/17	Total
First hospital MTC, n (%)	2736 (53%)	3885 (58%)	4813 (59%)	5496 (61%)	7078 (66%)	9322 (69%)	10,217 (69%)	11,468 (70%)	12,513 (72%)	67,528 (66.5%)
Time to arrival, hours, median (IQR)	1.2 (0.8–1.7)	1.2 (0.9–1.7)	1.2 (0.8–1.7)	1.2 (0.8–1.8)	1.3 (0.9–2)	1.5 (1.1–2.1)	1.6 (1.1–2.3)	1.7 (1.2–2.5)	1.7 (1.2–2.6)	1.5 (1–2.2)
CT at any time, n (%)	2690 (50%)	3766 (54%)	4874 (57%)	5954 (62%)	7371 (63%)	9748 (66%)	11,276 (69%)	12,818 (71%)	13,868 (72%)	72,365 (65%)
Seen by consultant in ED, n (%)	1504 (29%)	2103 (31%)	3183 (39.3%)	4250 (47%)	6169 (58%)	8103 (60%)	8963 (60%)	9876 (61%)	10,943 (63%)	55,094 (54%)
Intubated in ED, n (%)	701 (13.6%)	918 (13.6%)	1098 (13.6%)	1198 (13.3%)	1460 (13.7%)	1778 (13.2%)	1845 (12.4%)	1959 (12%)	1917 (11%)	12,874 (12.7%)
Treated at MTC, n (%)	3757 (73%)	5058 (75%)	6113 (75%)	6750 (75%)	8212 (77%)	10,790 (80%)	11,873 (80%)	13,279 (82%)	14,247 (82%)	80,079 (79%)
Blood given within 6 h n (%)	118 (2.2%)	270 (3.9%)	283 (3.3%)	259 (2.7%)	372 (3.2%)	391 (2.6%)	405 (2.5%)	470 (2.6%)	423 (2.2%)	2991 (2.7%)
TXA if blood given n (%)	0 (0%)	0 (0%)	7 (2.5%)	60 (23%)	236 (63%)	323 (83%)	365 (90%)	426 (91%)	382 (90%)	1799 (60%)
Time to surgery, median (IQR)	18 (5–50)	18 (5–46)	18 (6–45)	19 (6–46)	20 (7–45)	21 (9–47)	21 (10–48)	22 (11–47)	22 (10.9–49)	21 (7.8–47.1)
Admitted to ICU or HDU, n (%)	1656 (31%)	2288 (33%)	2719 (32%)	2982 (31%)	3101 (27%)	3696 (25%)	4151 (25%)	4638 (26%)	4595 (24%)	29,826 (27%)
LOS in hospital, median (IQR)	10 (5–21)	10 (5–21)	10 (5–19)	9 (5–18)	9 (5–19)	9 (5–18)	9 (5–19)	9 (5–19)	9 (5–19)	9 (5–19)
LOS in ICU/HDU, median (IQR)	4 (2–10)	4 (2–10)	4 (2–10)	3 (1–8)	3 (1–9)	3 (1–8)	3 (1–8)	3 (1–8)	3 (1–8)	3 (1–8)
Survival at discharge, n (%)	4891 (92%)	6313 (91%)	7895 (92%)	8808 (91%)	10,568 (90%)	13,388 (91%)	14,878 (91%)	16,424 (91%)	17,451 (91%)	100,616 (91%)
Time to death, median (IQR)	8 (5–14)	8 (4–14)	8 (4–13)	8 (4–13)	7 (4–13)	8 (4–13)	8 (4–14)	8 (4–14)	8 (4–14)	8 (4–14)

**Table 3:** Characteristics of the population from all hospitals by financial year 08/09–16/17 – patients with an ISS ≥ 9.

	Financial year									
	2008/09	2009/10	2010/11	2011/12	2012/13	2013/14	2014/15	2015/16	2016/17	Total
Number of hospitals submitting to TARN	91	120	149	164	175	176	173	171	169	189
Number of TARN cases that year	8903	12,123	17,956	23,211	28,239	33,647	37,725	42,371	44,059	248,234
Age, median (IQR)	47 (26–63.8)	51 (29–67)	52.6 (30–71)	55 (32–74)	58 (36–77)	60 (38–79)	62 (42–81)	63 (42.9–81.6)	64.5 (44–82)	59.6 (38–79)
Age > 64, n (%)	2041 (22.9%)	3288 (27.1%)	5400 (30.1%)	7752 (33.4%)	10,725 (38%)	14,005 (41.6%)	17,170 (45.5%)	20,147 (47.5%)	21,688 (49.2%)	102,216 (41.2%)
Male, n (%)	5798 (65.1%)	7772 (64.1%)	11,166 (62.2%)	14,217 (61.3%)	16,457 (58.3%)	19,323 (57.4%)	21,094 (55.9%)	23,775 (56.1%)	24,225 (55%)	143,827 (57.9%)
ISS, median (IQR)	10 (9–20)	12 (9–20)	11 (9–20)	10 (9–20)	10 (9–20)	10 (9–20)	10 (9–20)	10 (9–20)	12 (9–20)	10 (9–20)
First hospital GCS, median (IQR)	15 (14–15)	15 (14–15)	15 (14–15)	15 (14–15)	15 (15–15)	15 (15–15)	15 (15–15)	15 (15–15)	15 (15–15)	15 (15–15)
RTC, n (%)	2825 (31.7%)	3438 (28.4%)	4568 (25.4%)	5782 (24.9%)	6239 (22.1%)	7510 (22.3%)	8012 (21.2%)	8688 (20.5%)	8683 (19.7%)	55,745 (22.5%)
High fall, n (%)	1354 (15.2%)	1829 (15.1%)	2649 (14.8%)	3191 (13.7%)	3668 (13%)	4462 (13.3%)	4674 (12.4%)	4917 (11.6%)	5098 (11.6%)	31,842 (12.8%)
Low fall, n (%)	3279 (36.8%)	4948 (40.8%)	7984 (44.5%)	11,097 (47.8%)	14,973 (53%)	17,986 (53.5%)	21,271 (56.4%)	24,679 (58.2%)	25,869 (58.7%)	132,086 (53.2%)
Other, n (%)	676 (7.6%)	850 (7%)	1258 (7%)	1536 (6.6%)	1694 (6%)	1881 (5.6%)	1902 (5%)	2011 (4.7%)	2080 (4.7%)	13,888 (5.6%)
Assault, n (%)	769 (8.6%)	1058 (8.7%)	1497 (8.3%)	1605 (6.9%)	1665 (5.9%)	1808 (5.4%)	1866 (4.9%)	2076 (4.9%)	2329 (5.3%)	14,673 (5.9%)
Penetrating, n (%)	540 (6.1%)	689 (5.7%)	870 (4.8%)	1056 (4.5%)	989 (3.5%)	964 (2.9%)	975 (2.6%)	1199 (2.8%)	1330 (3%)	8612 (3.5%)
TBI, n (%)	2658 (30.6%)	3713 (31.5%)	5533 (32.3%)	6707 (30.5%)	7738 (29.3%)	9214 (29.6%)	10,272 (29.5%)	12,048 (30.8%)	12,753 (31.3%)	70,636 (30.5%)
Extracranial, n (%)	6527 (75.1%)	8761 (74.3%)	12,495 (73%)	16,321 (74.3%)	19,730 (74.8%)	23,199 (74.5%)	26,030 (74.7%)	28,753 (73.6%)	29,733 (73%)	171,549 (74%)
Comorbidity present, n (%)	3603 (40.5%)	5438 (44.9%)	8838 (49.2%)	12,510 (53.9%)	16,042 (56.8%)	20,121 (59.8%)	24,313 (64.4%)	29,367 (69.3%)	30,980 (70.3%)	151,212 (60.9%)

**Table 4:** Care process in all hospitals by financial year 08/09–16/17 – patients with ISS ≥ 9.

	Financial year									
	2008/09	2009/10	2010/11	2011/12	2012/13	2013/14	2014/15	2015/16	2016/17	Total
First hospital MTC, n (%)	2789 (32%)	4055 (34%)	5572 (32.6%)	6876 (31%)	9694 (36.8%)	12,588 (40%)	14,139 (40.6%)	15,694 (40%)	16,871 (41%)	88,278 (38%)
Time to arrival, median (IQR)	1.1 (0.8–1.6)	1.2 (0.9–1.7)	1.2 (0.8–1.8)	1.2 (0.8–1.8)	1.4 (1–2.1)	1.5 (1.1–2.2)	1.6 (1.2–2.4)	1.7 (1.2–2.6)	1.8 (1.3–2.8)	1.5 (1.1–2.3)
Intubated by Dr prehospital, n (%)	50 (0.6%)	80 (0.7%)	80 (0.5%)	41 (0.2%)	73 (0.3%)	80 (0.3%)	99 (0.3%)	73 (0.2%)	44 (0.1%)	620 (0.3%)
Arrival at first hospital midnight - 8:00 am, n (%)	1556 (17.5%)	2049 (16.9%)	2894 (16.1%)	3641 (15.7%)	4388 (15.5%)	5241 (15.6%)	5972 (15.8%)	6845 (16.2%)	7184 (16.3%)	39,770 (16%)
CT at any time, n (%)	4035 (45%)	5953 (49%)	8984 (50%)	12,313 (53%)	15,626 (55%)	19,774 (58.8%)	23,036 (61%)	27,059 (63.9%)	28,865 (65.5%)	145,645 (58.7%)
Seen by consultant in ED, n (%)	2188 (25%)	3218 (27.3%)	5217 (30.5%)	7601 (34.6%)	11,531 (43.7%)	14,406 (46.3%)	16,111 (46.3%)	17,691 (45.3%)	18,797 (46.2%)	96,760 (41.8%)
Seen by consultant in ED if ISS > 15, n (%)	1136 (31.9%)	1713 (34.6%)	2712 (38.2%)	3825 (43.7%)	5552 (54.8%)	7044 (57.7%)	7942 (57.8%)	8876 (56.4%)	9412 (56.8%)	48,212 (52%)
Seen by consultant in ED if GCS < 13, n (%)	459 (47.4%)	664 (52.2%)	1027 (58%)	1338 (62%)	1981 (72.9%)	2384 (75.4%)	2558 (74.6%)	2755 (74.8%)	2724 (76%)	15,890 (69.9%)
Intubated in ED, n (%)	951 (10.9%)	1248 (10.6%)	1639 (9.6%)	1898 (8.6%)	2386 (9%)	2700 (8.7%)	2850 (8.2%)	2976 (7.6%)	2929 (7.2%)	19,577 (8.4%)
Admitted direct or transfer to MTC, n (%)	3879 (44.7%)	5394 (45.7%)	7383 (43.1%)	8893 (40.5%)	11,803 (44.8%)	15,076 (48.4%)	16,837 (48.3%)	18,747 (48%)	19,811 (48.7%)	107,823 (46.5%)
Time to surgery, median (IQR)	19.9 (5.8–50.5)	19.4 (6.4–47.2)	19.35 (6.7–44.8)	20.5 (8.2–45.4)	20.4 (8.7–44)	21.5 (11.1–45.8)	22.1 (12.3–46)	22.5 (13.2–45.4)	23.3 (13.6–47.3)	21.7 (10.7–45.9)
Admitted to ICU or HDU, n (%)	2219 (24.9%)	3090 (25.5%)	4266 (23.8%)	5180 (22.3%)	5559 (19.7%)	6347 (18.9%)	7024 (18.6%)	7719 (18.2%)	7582 (17.2%)	48,986 (19.7%)
LOS in hospital, median (IQR)	10 (5–21)	10 (5–20)	9 (5–19)	9 (5–18)	9 (5–18)	9 (5–18)	10 (5–19)	10 (5–19)	10 (5–19)	10 (5–19)
LOS in ICU/HDU, median (IQR)	4 (2–9)	4 (2–9)	4 (2–9)	3 (1–7)	3 (1–8)	3 (1–7)	3 (1–7)	3 (1–7)	3 (1–7)	3 (1–8)
Survival at discharge, n (%)	8245 (92.6%)	11,129 (91.8%)	16,535 (92.1%)	21,385 (92.1%)	25,829 (91.5%)	30,808 (91.6%)	34,558 (91.6%)	38,733 (91.4%)	40,407 (91.7%)	227,629 (91.7%)
TXA given n (%)	2 (0%)	1 (0%)	24 (0.1%)	304 (1.3%)	1217 (4.3%)	2511 (7.5%)	3092 (8.2%)	3633 (8.6%)	3041 (6.9%)	13,825 (5.6%)
Blood given within 6 h n (%)	174 (2%)	333 (2.7%)	374 (2.1%)	396 (1.7%)	639 (2.3%)	633 (1.9%)	714 (1.9%)	810 (1.9%)	672 (1.5%)	4745 (1.9%)
TXA and blood given within 6 h n (%)	1 (0.6%)	1 (0.3%)	7 (1.9%)	89 (22.5%)	394 (61.7%)	485 (76.6%)	616 (86.3%)	717 (88.5%)	601 (89.4%)	2911 (61.3%)
Time to death within 30 days, median (IQR)	8 (5–14)	8 (4–14)	8 (4–13)	8 (4–13)	7 (4–13)	8 (4–13)	8 (4–14)	8 (4–14)	8 (4–14)	8 (4–14)

Of particular note is the 36.5% absolute increase in consultant attendance as trauma team leader (29% to 63% p < 0.0001) which was a key change made during trauma system reorganisation. Pre-hospital intubation is uncommon (overall 0.3%), and intubation rates in the ED have decreased from 13.6% to 11% (p < 0.0001). The administration of tranexamic acid in patients with haemorrhage requiring transfusion within 6 h of admission has significantly increased from near zero to 90% in 2016/17 (p < 0.0001). Median time to surgery (all types) has increased by 4 h (18 h to 22 h p < 0.0001). There has been a reduction in the proportion of patients requiring critical care (31% to 24% p < 0.0001) and in the median length of stay on critical care (4 to 3 days, p < 0.0001). Overall median length of stay in acute care was unchanged from initially 10 (IQR 5–21) to finally 9 (5 to 19) days. Similar trends in care processes were present in the sensitivity analysis which included patients from all hospitals (Table 4).

**Fig. 2:**
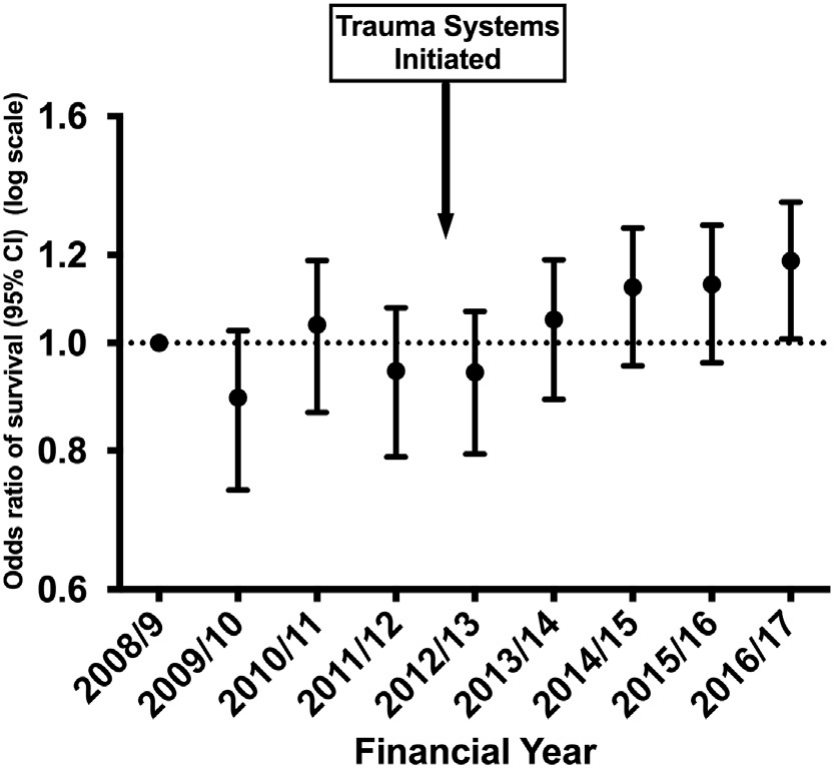
Trends in odds of surviving major trauma: April 2008–March 2017. Hospitals with consistent submissions. *ISS* ≥ 9, *missing GCS imputed*.

**Fig. 3:**
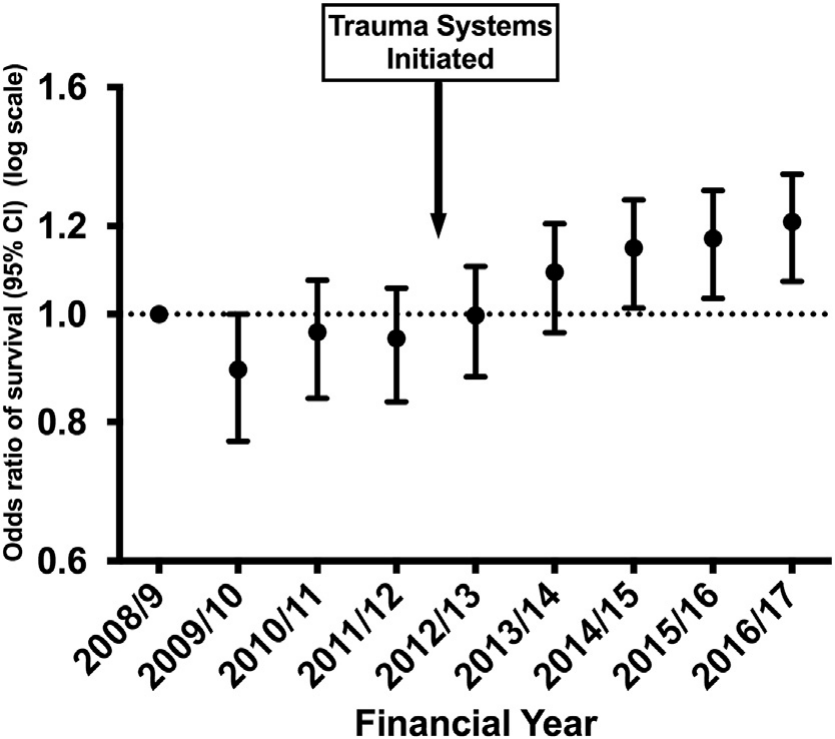
Trends in odds of surviving major trauma: April 2008–March 2017. All hospitals. *ISS* ≥ 9, *missing GCS imputed*.

There was no trend in the crude survival rate of 92% (chi square test for trend p = 0.052). The risk-adjusted survival for ‘consistent submitter’ hospitals (Fig. 2) showed an odds ratio of 1.19 (95% CI 1.03 to 1.36) for survival comparing 2008/09 and 2016/17. The trend for the 0.19 (95% CI 0.03–0.36) increase in adjusted odds is significant (p = 0.012). The same pattern is seen in the sensitivity analysis of patients from all hospitals, with an odds ratio of 1.21 (95% CI 1.08 to 1.35) (Fig. 3). The Area under the Receiver Operator curve for the model used in risk adjustment was 0.896 (95% CI 0.893–0.898). In the adjustment model 20,852 patients (8.4%) had missing data imputed for GCS (missing GCS being imputed using the conventional TARN method [17]) and 28,768 had missing data for comorbidities (with ‘Missing’ used as category in the model). Further sensitivity analyses on cases with complete data (excluding those with missing data) showed similar increases in risk adjusted odds of survival over time (not shown).

**Fig. 4:**
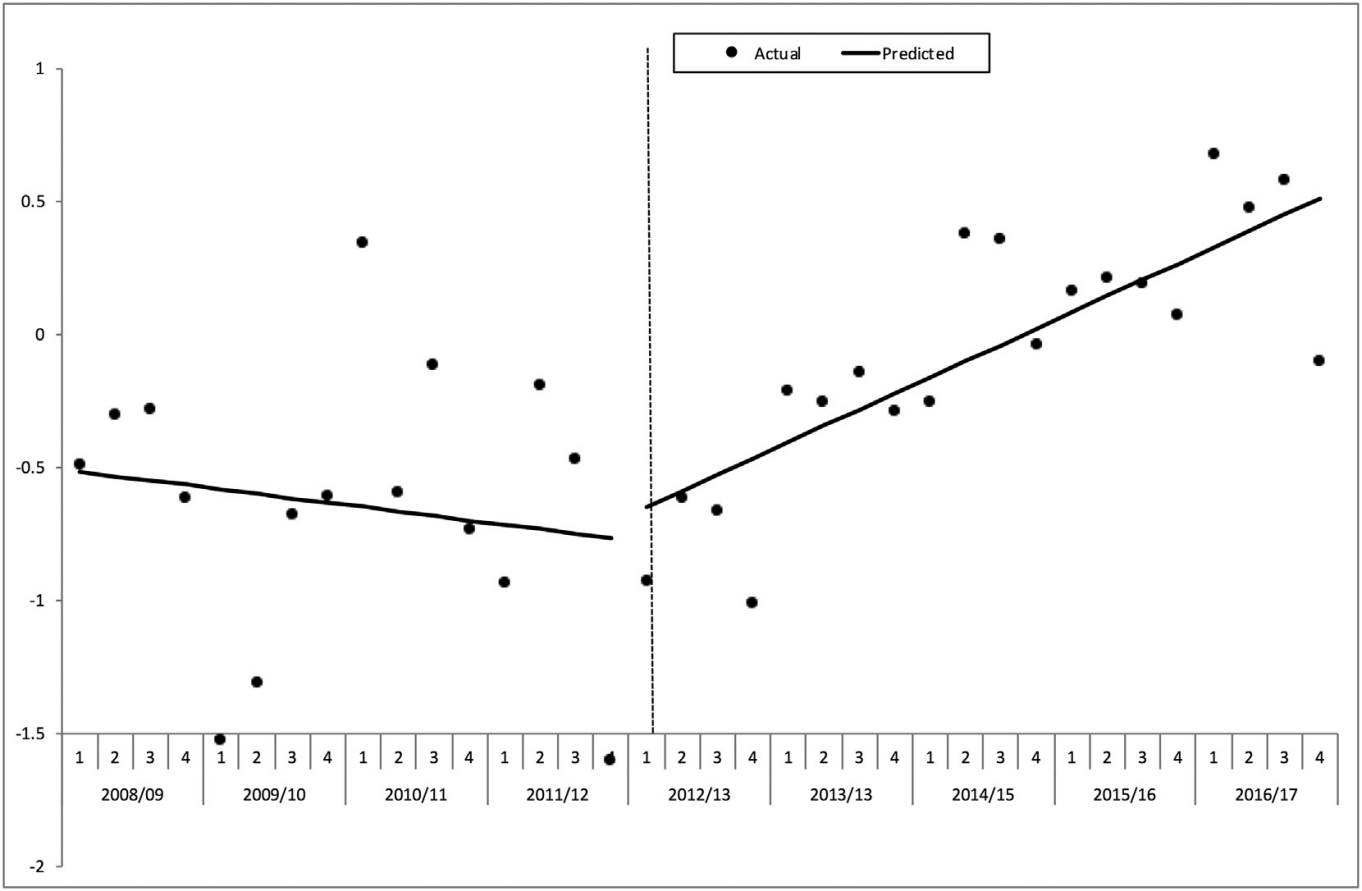
Interrupted times series analysis of change in excess survival rate per 100 patients (W) around intervention in financial year 2012/13. Hospitals with consistent submissions. *ISS* ≥ 9, *missing GCS imputed*.

The interrupted time series analysis of quarterly “W” for the ‘consistent submitters’ (Fig. 4) showed that prior to the intervention there was no significant change with time (a slope of − 0.02% excess survivors per quarter, p = 0.526). Around the time of interruption in the time series there was no significant change in the level of the line (+ 0.133% excess survivors, p = 0.678). However, there was a significant positive change in the slope after the intervention time point of April 2012 (+ 0.08% excess survivors per quarter, p = 0.023), in other words an increase of 0.08 more survivors per 100 patients every quarter.

**Fig. 5:**
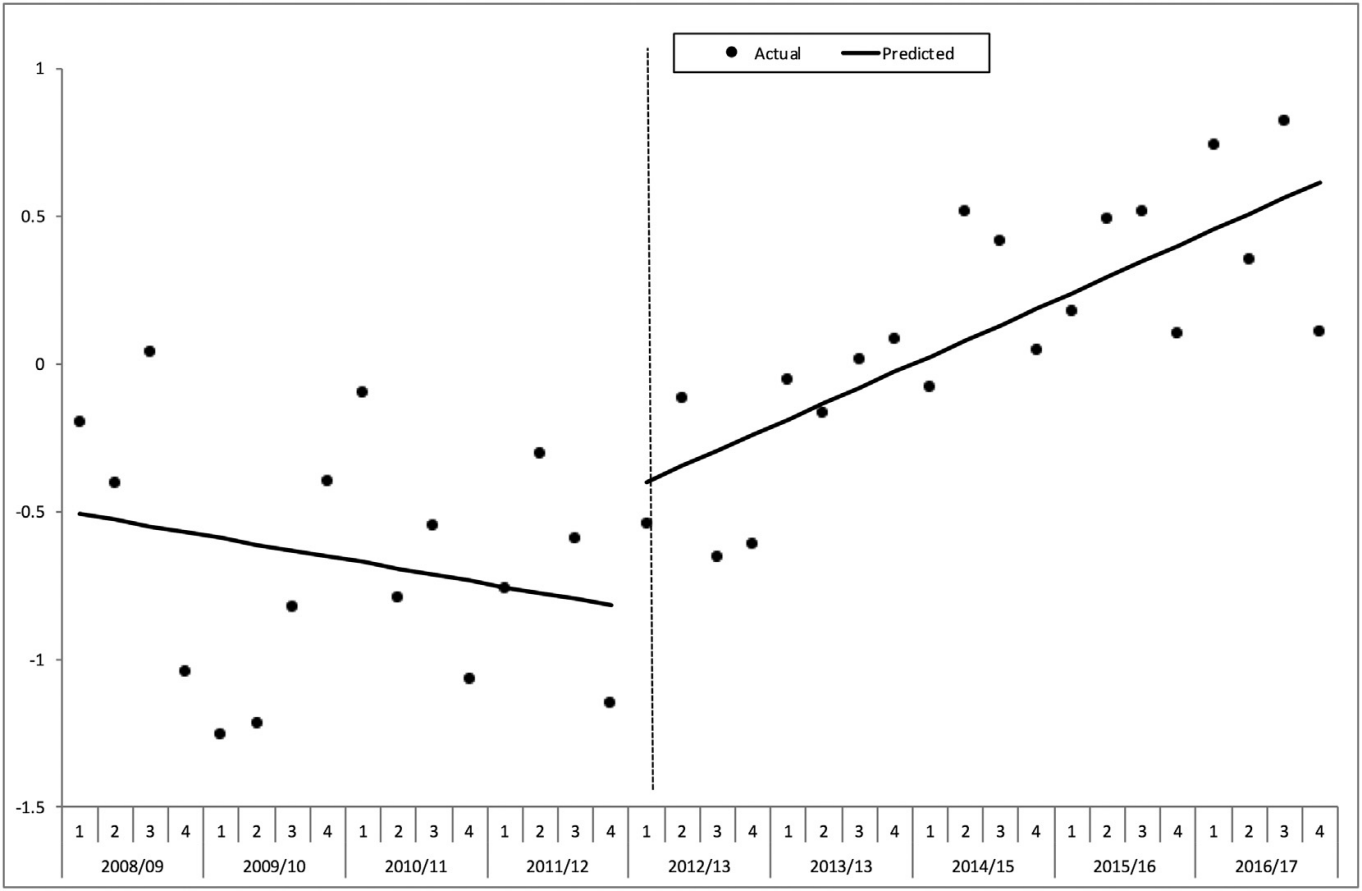
Interrupted times series analysis of change in excess survival rate per 100 patients (W) around intervention in financial year 2012/13. All hospitals. *ISS* ≥ 9, *missing GCS imputed*.

The sensitivity analysis using all eligible patients in the TARN database rather than just those from the hospitals who were ‘constant submitters’ (Fig. 5) showed the same pattern of a positive change after intervention in the slope of “W” with time (+ 0.07% excess survivors per quarter, p = 0.006). The same pattern was again seen when the analysis was restricted to only those cases with complete data (not shown).

## 5 Discussion

We have described the changes in the process of care that happened during the implementation of Major Trauma Systems in England and also the changing demographic of trauma in the population. Our analysis of a cohort of rigorously identified and described major trauma patients indicates that these changes have been associated with a significant 19% increase in the risk adjusted odds of survival for trauma victims who reach hospital alive. Experience in other regional systems, such as Victoria State, Australia, suggests that improvements in mortality may continue for up to five years as a new trauma system matures [8].

It is not possible to study a complete change in a national system of care with a randomised controlled trial and so we have performed a longitudinal, series of annual cross sectional observational studies using continuous data collection, which attempts to cover the entire national population. The statistical methodology has been tested and refined over many years of national trauma audit, and allows risk adjustment for the observed changes in case-mix over time. A longitudinal study is the best method available, however the limitation remains that controlling for all known and unknown confounding factors in this large, population-based study is not possible. There remains the potential for confounding from changes in data collection, as introducing the new system appears to have resulted in a significant increase in data collection with all eligible hospitals in England now participating in national trauma audit.

There has been a change in recorded demographics with a significant increase in the age of patients and more patients injured in falls from less than 2 m. This change is likely due to the increasing age of the population in England together with consistent under-reporting of trauma in older patients until the new networks commenced. Data collection was incomplete, with particular difficulty in obtaining comprehensive data from Trauma Units. Notwithstanding the increase the number of hospitals submitting data to TARN, the numbers of TARN cases per annum in the 35 hospitals in the main study trebled over the study period, and cases per hospital per annum in the ‘all patients’ dataset doubled. TARN assesses case ascertainment against Hospital Episode Statistics (HES) trauma codes in hospitalised patients meeting the inclusion criteria. The numbers of patients coded as major trauma under HES in the consistently submitting hospitals has increased by 117% over the study period. There have also been changes in clinical practice during the study period. Massive transfusion protocols [21], [22], [23], the use of Tranexamic Acid [24], [25] and whole body (trauma) CT scan [26] have a good evidence-base and have all become routine clinical practice during resuscitation for major trauma during this study.

In comparison with previous studies, we have taken a national perspective and have included major trauma patients who are treated outside of major trauma centres as well as those treated at the centres of excellence. This is important as it is certainly possible that system changes which benefit one group of patients (those who get to a MTC) might disadvantage another group of patients (those who are treated outside an MTC). Many previous studies have just taken the MTC perspective and not included all patients [6]. Our results are similar to those found in the Australian state of Victoria, where a similar comprehensive view was taken [8].

This study supports the healthcare policy of regionalisation of specialist services for the care of major trauma that has been implemented within NHS-England. It is not possible to determine the specific cause of changes in outcome as there are complex system changes taking place, however it looks as if the overall result of the changes is an improvement in outcomes. The exact cause of better outcomes cannot be determined, and it is likely that the outcomes were influenced by a complex combination of the changes in patient flow, the changes in access to investigations and interventions, the changes in the seniority of personnel available and the changes in the philosophy of care. In such a complex intervention there may be little benefit in attempting to identify a simple change/outcome relationship. As well as the changes in the organisation of trauma services there were also significant changes in clinical care during the study (such as the increased use of tranexamic acid following from the CRASH2 trial). However, the trauma care reorganisation may have also enabled the implementation of improvements in clinical care. It is recognised that innovative change in clinical practice, even when supported by level-1 evidence, can be slow to gain acceptance [27]. Transition from research to routine clinical practice can be extremely slow and so it is unlikely that these changes in practice would have been implemented so quickly and so universally had the organisation of trauma care not changed. This may have been because the MTCs all adopted similar treatment protocols, largely based upon the translation into civilian practice of the military experience in Iraq and Afghanistan [28].

There are still a large number of unanswered questions. Whilst regionalised care is associated with an overall benefit, there may be specific patient subgroups, such as infants or older patients, who have a different way of presenting to healthcare after major injury and may be disadvantaged. A number of decisions, based on clinical consensus, were made in the development of the English trauma systems (such as the bypass radius for each MTC), and further evidence is required to optimise system structures.

## 6 Conclusion

A change in the organisation of care for patients with severe injuries, including the development of Major Trauma Networks that cover the entire national population, was associated with a significant 19% (95% CI 3%–36%) increase in the odds of survival for trauma victims who reach the hospital alive (p = 0.012).

## Copyright statement

The Corresponding Author has the right to grant on behalf of all authors and does grant on behalf of all authors, a worldwide licence to the Publishers and its licensees in perpetuity, in all forms, formats and media (whether known now or created in the future), to i) publish, reproduce, distribute, display and store the Contribution, ii) translate the Contribution into other languages, create adaptations, reprints, include within collections and create summaries, extracts and/or, abstracts of the Contribution, iii) create any other derivative work(s) based on the Contribution, iv) to exploit all subsidiary rights in the Contribution, v) the inclusion of electronic links from the Contribution to third party material where-ever it may be located; and, vi) licence any third party to do any or all of the above.

## Competing Interests Statement

All authors have completed the ICMJE uniform disclosure form at www.icmje.org/coi_disclosure.pdf and declare: no support from any organisation for the submitted work; CM and KW are employed by NHS England and are responsible for the implementation of trauma networks in England and Wales; no other relationships or activities that could appear to have influenced the submitted work. TARN is based within a University Department and is funded through subscriptions from the hospitals that take part in the audit.

## Authorship Statement

All of the authors were involved in each stage of the planning, interpretation of results and write up of the work. The original plan was developed by CM, FL, MW, TC and KW. The statistical analysis was performed by OB and TL, with all authors taking part in the interpretation of the results. The manuscript was drafted by CM, FL, OB and TC with all authors commenting on the draft versions. The final version has been approved by CM, FL, OB, TL, AE, KW and TC.

## Contribution Statement

MW, FL, OB, AE and TC have been involved in the assessment of the quality of trauma care through the Trauma Audit and Research Network for many years and have developed the standard methodologies used in this work (MW as TARN Chief Executive, FL as TARN Director of Research, OB as the TARN Statistician and TC as Chair of the TARN Board). The original idea for the work came from the NHS England Clinical Advisory Group in discussions held between 2008 and 2010. The plan for the study was developed by CM, FL, MW, TC and KW. The statistical analysis plan was developed between CM, FL, OB, TL and KW and was carried out by OB. All of the authors were involved in discussion about the interpretation of the results and the best method of presentation.

## Data Sharing Statement

Data sharing: patient level data are available, subject to a standard data sharing agreement (which can be found at www.tarn.ac.uk), from the corresponding author at chris.moran@nuh.nhs.uk. Individual participant consent for data sharing was not obtained, but the presented data are anonymised, risk of identification is low and approval is in place from the UK Department of Health's Patient Information Advisory Group.

## Transparency Declaration

The lead author Prof Chris Moran is the manuscript's guarantor who affirms that the manuscript is an honest, accurate, and transparent account of the study being reported; that no important aspects of the study have been omitted; and that any discrepancies from the study as planned have been explained.

## Acknowledgements/Funding

This analysis was carried out independently and did not receive funding. The data collection for the national clinical audit was funded by subscriptions from participating hospitals, who did not have any role in study design, data analysis, interpretation, writing of the report.
